# Lens metabolomic profiling as a tool to understand cataractogenesis in Atlantic salmon and rainbow trout reared at optimum and high temperature

**DOI:** 10.1371/journal.pone.0175491

**Published:** 2017-04-18

**Authors:** Sofie Charlotte Remø, Ernst Morten Hevrøy, Olav Breck, Pål Asgeir Olsvik, Rune Waagbø

**Affiliations:** 1 National Institute of Nutrition and Seafood Research (NIFES), Bergen, Norway; 2 Marine Harvest ASA, Bergen, Norway; Universidade de Vigo, SPAIN

## Abstract

Periods of high or fluctuating seawater temperatures result in several physiological challenges for farmed salmonids, including an increased prevalence and severity of cataracts. The aim of the present study was to compare cataractogenesis in Atlantic salmon (*Salmo salar* L.*)* and rainbow trout (*Oncorhynchus mykiss*) reared at two temperatures, and investigate whether temperature influences lens metabolism and cataract development. Atlantic salmon (101±2 g) and rainbow trout (125±3 g) were reared in seawater at either 13°C (optimum for growth) or 19°C during the 35 days experiment (n = 4 tanks for each treatment). At the end of the experiment, the prevalence of cataracts was nearly 100% for Atlantic salmon compared to ~50% for rainbow trout, irrespective of temperature. The severity of the cataracts, as evaluated by slit-lamp inspection of the lens, was almost three fold higher in Atlantic salmon compared to rainbow trout. The global metabolic profile revealed differences in lens composition and metabolism between the two species, which may explain the observed differences in cataract susceptibility between the species. The largest differences were seen in the metabolism of amino acids, especially the histidine metabolism, and this was confirmed by a separate quantitative analysis. The global metabolic profile showed temperature dependent differences in the lens carbohydrate metabolism, osmoregulation and redox homeostasis. The results from the present study give new insight in cataractogenesis in Atlantic salmon and rainbow trout reared at high temperature, in addition to identifying metabolic markers for cataract development.

## Introduction

Cataract is characterized as the presence of opacities in the eye lens, which can be caused by changes in the epithelial tissues surrounding the lens fibres, or the composition and structure of the lens fibres, resulting in reduced vision [1]. The aetiology of cataract in farmed fish species is considered to be production related; caused by both sub-optimal environmental conditions and nutritional deficiencies [1].

During the summer months, periods of naturally high or fluctuating sea water temperatures may cause unfavourable conditions for farmed salmonids, resulting in several physiological and fitness consequences [2,3,4,5,6]. In a study by Waagbø et al. [7] a natural increase in seawater temperatures (in the range 12–18.5°C) resulted in a higher prevalence of cataracts in adult Atlantic salmon during the second year in sea. The underlying causes for the increased cataract development at high temperature have not been established, however, increased oxidative pressure and an alteration in nutrient requirement due to changes in the metabolism and growth rates are hypothesized to promote cataract development [7]. Water temperature affects the growth rate and metabolism of poikilotherm animals, and the growth of salmon post-smolt increases up to the optimum temperature of 13°C [8]. Previous studies have identified that fluctuating water temperatures, although at temperatures below the optimum for growth (range 2–8°C) increased cataract development in smolting Atlantic salmon [9]. Also, several studies have shown a correlation between rapid growth rates and cataract development [10, 11, 12]. At temperatures above the optimum for growth, the metabolism and energy demand for maintenance increases [13]. Oxidative damage to proteins, lipids and DNA can result from high concentrations of free radicals and their reactive non-radical derivatives that are produced by the normal metabolism [14]. The decreased protein turnover towards the lens native nucleus makes the lens tissue especially vulnerable to increased ROS production in the epithelial cells [15]. Exposure to high temperatures has been shown to result in a higher oxidative pressure and alterations in the integrated cellular antioxidant defence system in goldfish (*Carassius auratus*) tissues [16, 17].

Historically, cataracts have been observed in both Atlantic salmon and rainbow trout, and have been associated with sub-optimal levels of methionine, tryptophan, riboflavin, zinc and manganese [1]. However, in recent years, a high prevalence of cataracts have especially been reported in farmed Atlantic salmon, in relation to low dietary levels of the essential amino acid histidine [7, 18, 19]. In periods when the fish is sensitive, such as after seawater transfer and in periods with high or fluctuating water temperatures, the requirement for histidine to reduce the risk of cataract development appears to be higher than the estimated requirement for growth in Atlantic salmon [7, 19, 20]. Histidine is taken up by the lens and synthesized to N-acetylhistidine (NAH), and the severity of cataracts is negatively correlated with the concentration of NAH in the lens [7, 19, 20]. Lens NAH has been shown to have important roles as an osmolyte [21, 22], buffer component [19] and possibly intracellular antioxidant [23], and is therefore essential in maintaining the lens water balance and cell integrity. Oxidative stress plays an important role in the development of cataracts in both animals and humans [24, 25], and a balanced pro- and antioxidant level in the diet has been shown to reduce cataract prevalence in Atlantic salmon [26].

The aim of the present study was to compare cataract development in Atlantic salmon and rainbow trout reared at optimal and high seawater temperature, and investigate whether rearing temperature influence lens metabolism and cataract development. The global metabolic profile was determined in individual lenses from both species with defined cataract scores, to investigate whether the lens composition or metabolism could give further insight in the cataractogenesis in salmonids. Analysis of the metabolome has been shown to be a useful tool in ophthalmological research [27, 28, 29], and to describe the effect of temperature on the metabolism of fish, in tissues [30] and plasma [6].

## Material and methods

The experiment complied with the guidelines of the Norwegian Regulation on Animal Experimentation and EC Directive 86/609/EEC, and the protocol was approved by the competent person at the laboratory unit at the Institute of Marine Research, Matre, Norway (FOTS licence #110), and the National Animal Research Authority. At the time the experiment was done, it was not necessary to obtain specific approval for animal experiments where the fish were not subjected to un-physiological conditions, according to the Norwegian legislation on experiments with animals (FOR-1996-01-15-23). The fish were reared at two temperatures that are within the tolerable range for Atlantic salmon and rainbow trout, and no pain and suffering was expected. Fish health and welfare was monitored during the experiment, as well as environmental conditions and feed intake. The behaviour of the fish was normal, and plasma cortisol (as an indication of stress) was not elevated. There was no mortality during the experiment. According to the legislation, all the involved scientists are licensed as FELASA (Federation of Laboratory Animal Science Associations) C researcher.

### Experimental fish

The experiment was carried out at Matre Aquaculture Research Station (Matredal, Norway), from November 10^th^ to December 15^th^, 2010. Atlantic salmon (autumn transfer post-smolt, body weight 101±2 g) and rainbow trout (125±3 g), were randomly distributed in 16 tanks (1 m^3^), eight tanks for each species (n = 80), with a continuous flow of seawater (salinity 35 g L^-1^), 18:6 light regime and the addition of oxygenated water to keep the saturation at stable levels above 90%. The fish was acclimatized for three weeks at 13°C before the experiment start, and all groups were given the same experimental diet, prepared to be commercially relevant by Skretting, Norway (Table 1). The amino acid composition met the amino acid requirement for both species (NRC, 2011). The feed was fed in excess three times per day by the use of automatic feeders (ARVO-TEC T Drum 2000, Arvotec, Huutokoski, Finland) and uneaten pellets collected 15 min after each meal. During the first six days of the experiment the temperature was raised to 19°C in eight of the 16 tanks (1°C day^-1^), giving four tank replicates for each species on each temperature and the fish was reared on 19°C or 13°C for the remainder of the 35 day experiment.

**Table 1:** Feed ingredients and analysed feed composition of the experimental diets.

**Feed ingredients**	
Fish meal LT Scandinavian (Welcon AS) (g kg^-1^)	150
Soya protein concentrate (IMCOPA International S/A) (g kg^-1^)	139
Corn gluten (Cargill Nordic A/S) (g kg^-1^)	150
Wheat gluten (Cargill Nordic A/S) (g kg^-1^)	250
Wheat (Raffeissen Hauptgenossen Nord) (g kg^-1^)	40
North Atlantic fish oil (Triple Nine Fish Protein Amba) (g kg^-1^)	229
Vitamin and mineral premix (Trouw Nutrition, Putten, The Netherlands) (g kg^-1^)	42
Astaxanthin 10% (Nutritional Ingredients BV) (g kg^-1^)	0,5
**Analysed feed composition**	
Protein (g kg^-1^)	509
Lipid (g kg^-1^)	280
Dry matter (g kg^-1^)	950
Ash (g kg^-1^)	50
Energy (MJ kg^-1^)	25
Histidine (g kg^-1^)	10
Vitamin C (mg kg^-1^)	96

### Sampling procedures

Tissue sampling and cataract evaluation was performed four hours post-prandially. At the time of sampling, the fish were sedated by placing them in a solution of tricaine methanesulfonate (70 mg L^-1^) in seawater, and killed with a single blow to the head before dissection. Cataract assessment was performed on both lenses from each fish on day 0 (n = 30 fish for each species) and day 35 (n = 4 tanks; each of 9 examined fish) of the experiment with a Kowa SL-15 slit-lamp biomicroscope (Kowa, Tokyo, Japan) under darkened conditions. Each lens was given a score of 0 to 4, indicating the degree of opacification, giving a total score of 0–8 per fish, based on the method described by Wall and Bjerkås [31]. Cataracts are reported as prevalence (% of fish affected) and the mean cataract score for each experimental group. Lenses were carefully dissected from six fish from each species at day 0 (n = 12) and from three fish from each tank on day 5 and 35 (n = 6 from each tank, 24 lenses from each experimental group). The lenses were gently rolled on soft paper to remove aqueous humour, put into separate pre-tared tubes, immediately frozen on dry ice and stored at -80°C until determination of NAH and total free amino acid concentration (n = 6 per experimental group), and the global metabolic profile (n = 12 per experimental group).

### Sample preparation and metabolic profiling

Metabolic profiling analysis was performed by Metabolon as previously described [32,33]. A total of 48 lenses, 12 from each experimental group and each with a defined cataract score were analyzed. Briefly, samples were prepared using the automated MicroLab STAR^®^ system from Hamilton Company. A recovery standard was added prior to the first step in the extraction process for quality control (QC) purposes. Sample preparation was conducted using aqueous methanol extraction process to remove the protein fraction while allowing maximum recovery of small molecules. The resulting extract was divided into four fractions: one for analysis by UPLC/MS/MS (positive mode), one for UPLC/MS/MS (negative mode), one for GC/MS, and one for backup. The LC/MS portion of the platform was based on a Waters ACQUITY ultra-performance liquid chromatography (UPLC) and a Thermo-Finnigan linear trap quadrupole (LTQ) mass spectrometer, which consisted of an electrospray ionization (ESI) source and linear ion-trap (LIT) mass analyzer. The MS analysis alternated between MS and data-dependent MS^2^ scans using dynamic exclusion. The samples destined for GC/MS analysis were re-dried under vacuum desiccation for a minimum of 24 hours prior to being derivatised under dried nitrogen using bistrimethyl-silyl-triflouroacetamide (BSTFA). The GC column was 5% phenyl and the temperature ramp was from 40° to 300°C in a 16 minute period. Samples were analyzed on a Thermo-Finnigan Trace DSQ fast-scanning single-quadrupole mass spectrometer using electron impact ionization. Raw data was extracted, peak-identified and QC processed using Metabolon’s hardware and software. Metabolites were identified by automated comparison of the ion features in the experimental samples to a reference library of chemical standard entries that included retention time, molecular weight (*m/z*), preferred adducts, and in-source fragments as well as associated MS spectra [34].

### Quantification of lens free amino acid and imidazole composition

N-acetylhistidine (NAH) in the lens was determined with reverse phase HPLC (Waters Corporation, Milford, MA, USA) [35], slightly modified [19]. From the same lens extract, total free amino acid composition was determined by ninhydrin detection with Biochrom 20 plus (Biochrom Ltd, Cambridge, UK), by applying the Amino analyzer ninhydrin method (Amersham Pharmacia Biotech, Sweden) [19].

### Calculations and statistics

Statistical analysis was performed with Statistica software (Statsoft Inc., Tulsa, OK, USA) on replicate tank level (n = 4 tanks per experimental group) in a factorial design consisting of two species reared at two temperatures). Cataract prevalence, score and quantified analytical results are presented as mean ± SEM. Levene’s test was performed to test for homogeneity of variances. A confidence level of 95% was used for all the tests, giving a probability level of 0.05. A one-way ANOVA was performed to investigate the differences between species at the start of the experiment. A two-way ANOVA was performed to investigate the effect of temperature vs. species differences. Tukey’s HSD *post hoc* test was performed when the ANOVA results were significant (p<0.05). Graphpad prism version 6.0 (GraphPad Software, Inc. La Jolla, CA, USA) was used to create the figures.

For statistical analyses and data display purposes of the lens global metabolic profile, any missing values were assumed to be below the limits of detection and these values were imputed with the compound minimum (minimum value imputation). The raw data for each biochemical was re-scaled to have a median equal to 1. Statistical analysis of log-transformed data was performed using “R” (http://cran.r-project.org/), which is a freely available, open-source software package. A two-way ANOVA with contrasts was used to identify statistically significant (p≤0.05) species effects, temperature effects, and species by temperature interactions. A one-way ANOVA was used to investigate the effects of cataract score on the global metabolic profile of lenses for each species. Multiple comparisons were accounted for by estimating the false discovery rate (FDR) using q-values. The metabolites are shown as mean (+) and median (-) value, upper and lower quartiles, max and min of distributions (n = 12). Extreme data points are indicated by a circle (o).

## Results

### Experimental fish

During the 35 days long experiment both species grew well, and there was no mortality [3]. The rainbow trout had a higher feed intake and growth rate compared to the Atlantic salmon, however no differences were found within the species as a result of different temperatures. The mean end weight was 196 ± 4 g (SD) for Atlantic salmon and 293 ± 8 g for rainbow trout [3]

### Cataract prevalence and severity

**Fig 1:**
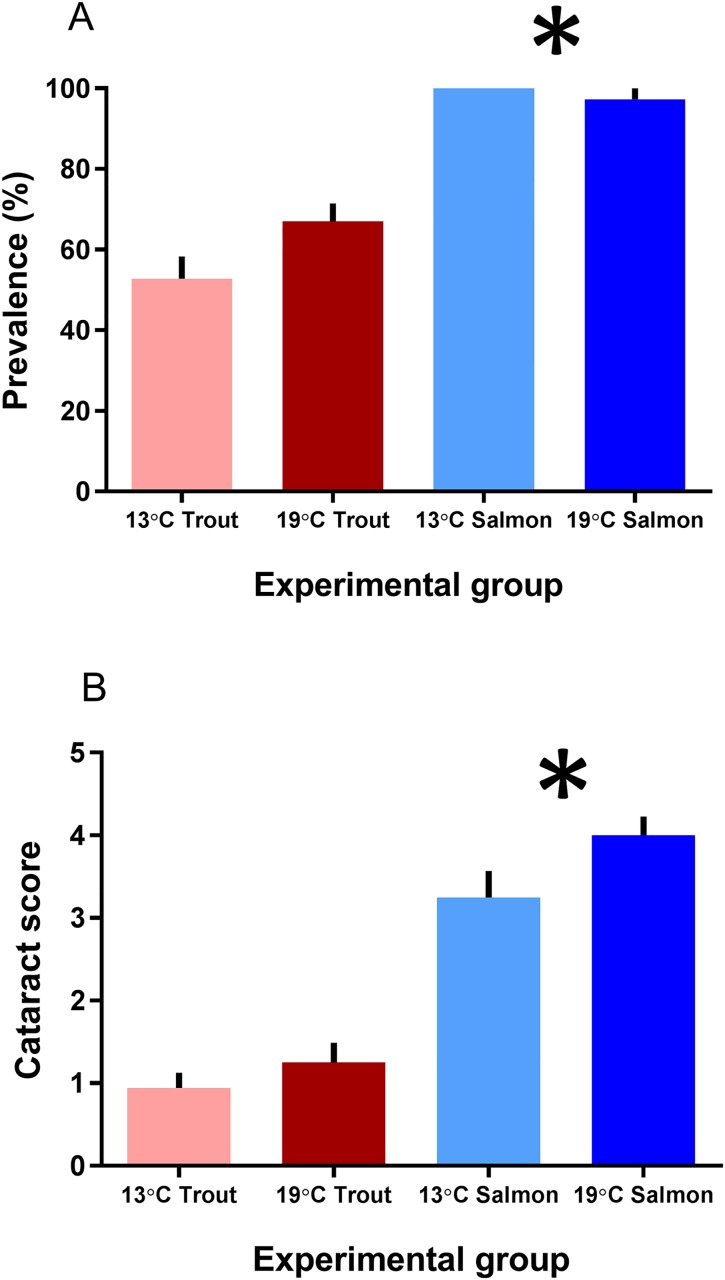
Cataract prevalence and severity. (A) Cataract prevalence and (B) severity (evaluated as score 0–4 on each lens) in lenses from Atlantic salmon and rainbow trout reared at 13 or 19°C at the end of the 35d experiment, as mean ± SEM (n = 4). Significant differences between the species are denoted by an asterisk (*) (p<0.05).

At the start of the experiment, the Atlantic salmon had a cataract prevalence of 70% and the cataract score was low grade with mean 1.3 ± 0.2 (n = 30). At the end of the 35 days experiment all of the Atlantic salmon reared at 13°C had developed cataracts (prevalence of 100%), and the severity had increased to a mean cataract score of 3.2 ± 0.3 (n = 4 tanks; each of 9 examined fish) (Fig 1). The prevalence of cataracts in Atlantic salmon reared at 19°C was 97 ± 2%, and the mean cataract score was 4.0 ± 0.2 (n = 4 tanks; each of 9 examined fish). Rearing temperature had no effect on the final prevalence and severity of cataracts. The rainbow trout had a cataract prevalence of 10% at the start of the experiment (n = 30), and the mean cataract score was 0.10 ± 0.06. At the end of the experiment the prevalence of cataract was 53 ± 5% and 67 ± 4% in rainbow trout reared at 13°C and 19°C, respectively (p = 0.09, n = 4 tanks; each of 9 examined fish) (Fig 1). The mean cataract score was equal in the 13°C and 19°C groups, with 0.9 ± 0.2 and 1.3 ± 0.2, respectively (n = 4 tanks; each of 9 examined fish).

### Lens biochemicals and metabolites

A total of 192 biochemicals were identified in the lenses (S1 Table). Of these, 125 were present at significantly different levels in Atlantic salmon compared to rainbow trout. Elevated rearing temperature affected the level of 75 of the identified biochemicals, and the changes were mostly similar between the two species. A relation between increasing cataract score and changes in the biochemical level was found for 15 biochemicals in Atlantic salmon lenses (S2 Table), and five biochemicals in rainbow trout lenses (S3 Table).

**Fig 2:**
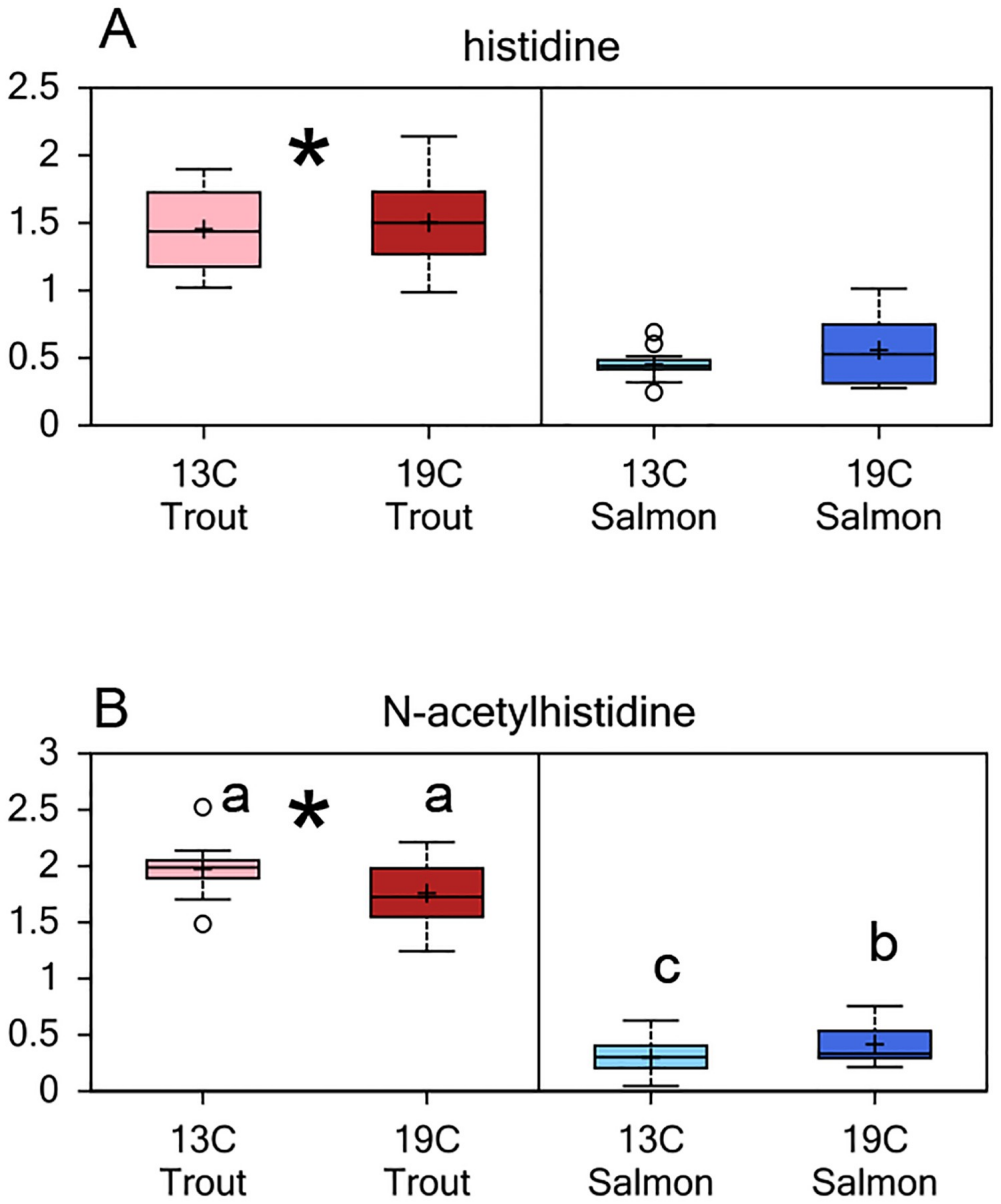
Lens histidine and N-Acetylhistidine (NAH) levels. (A) Relative levels of histidine and (B) NAH in lenses from Atlantic salmon and rainbow trout reared at 13 or 19°C at the end of the 35d experiment. Significant differences between the species are denoted by an asterisk (*), significant differences between temperatures and interaction effects are denoted by lower case letters (a, b) (p<0.05).

**Fig 3:**
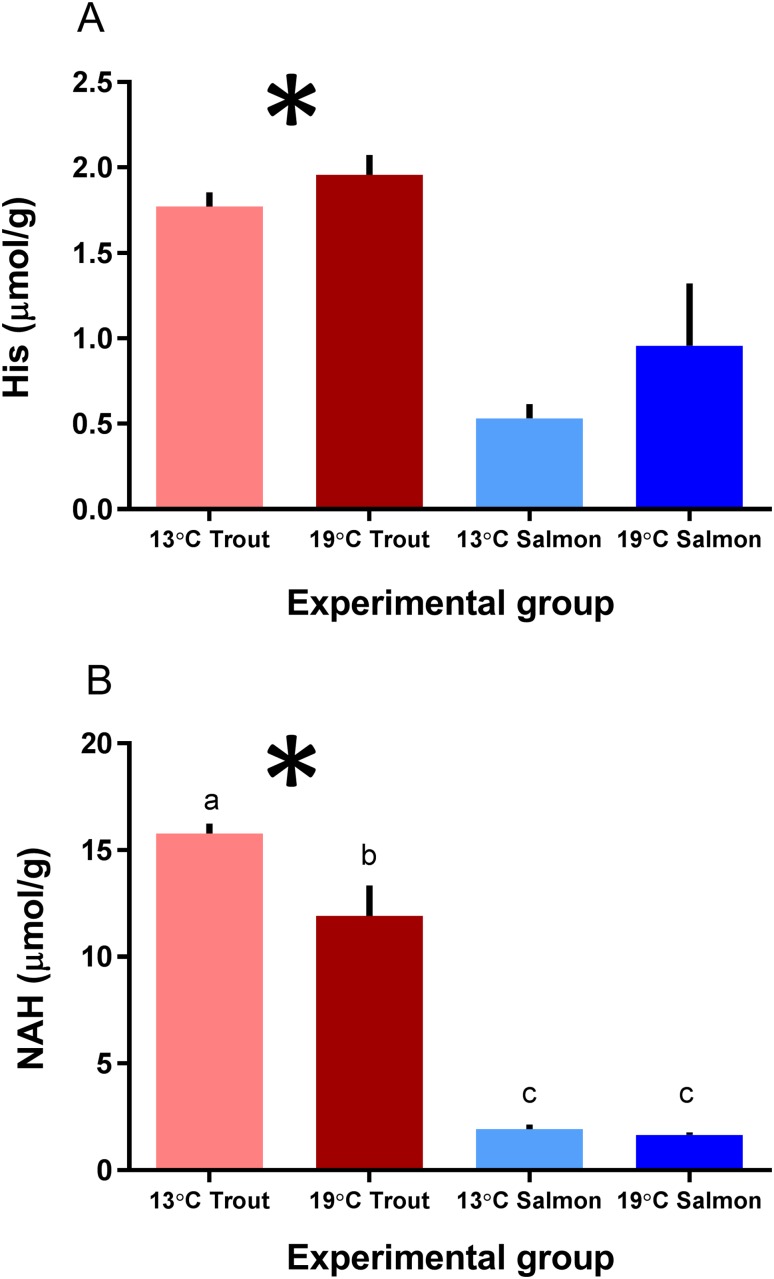
Lens histidine and N-Acetylhistidine (NAH) concentrations. (A) Lens histidine and (B) NAH concentrations in lenses from Atlantic salmon and rainbow trout reared at 13 or 19°C at the end of the 35d experiment, as mean ± SEM (n = 4). Significant differences between the species are denoted by an asterisk (*), significant differences between temperatures and interaction effects are denoted by lower case letters (a, b) (p<0.05).

The global metabolic profile showed that the metabolism of amino acids differed between the species (S1 Table), whereas a major difference was in the histidine metabolism, with significantly higher levels of both histidine and NAH in rainbow trout lenses compared to Atlantic salmon (Fig 2). A separate quantification of lens histidine and NAH levels showed that at the start of the experiment (d0) the lens free histidine concentration was approx. three-fold higher in rainbow trout compared to Atlantic salmon, with mean concentrations of 2.9 ± 0.2 and 1.0 ± 0.1 μmol g^-1^, respectively. At the end of the experiment (d35) the lens free histidine concentration was reduced in both species, but was still significantly higher in rainbow trout compared to Atlantic salmon, irrespective of rearing temperature, with mean concentrations of 1.9 ± 0.1 and 0.7 ± 0.2 μmol g^-1^, respectively (Fig 3). Initially, the rainbow trout had approx. nine-fold higher lens NAH concentration compared to Atlantic salmon, with a mean concentration of 12.4 ± 2.1 compared to 1.4 ± 0.2 μmol g^-1^. The NAH concentration remained at a similar low concentration during the experiment in the Atlantic salmon lenses, irrespective of rearing temperature. At the end of the experiment the lens NAH concentration was significantly lower in rainbow trout reared at 19°C compared to 13°C, with mean concentrations of 11.9 ± 1.4 and 15.8 ± 0.5 μmol g^-1^, respectively (Fig 3).

**Fig 4:**
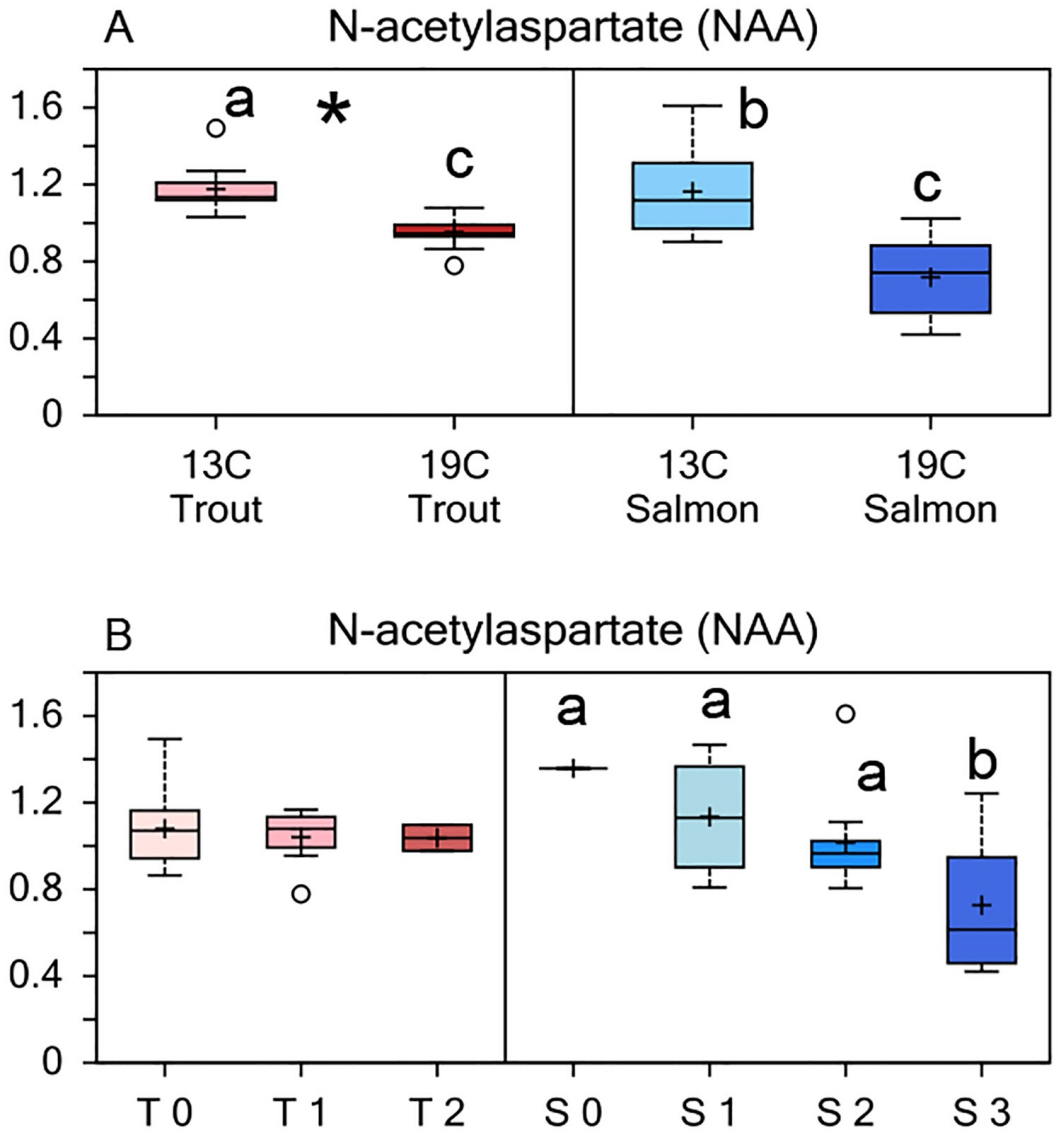
Lens N-Acetylaspartate (NAA) levels. (A) Relative levels of NAA in lenses from Atlantic salmon and rainbow trout reared at 13 or 19°C at the end of the 35d experiment, and (B) NAA levels in relation to cataract score (mean level for lenses scored 0–3 for each of the species, T: trout, S: salmon) Significant differences between species are denoted by an asterisk (*), significant differences between temperatures and interaction effects are denoted by lower case letters (a, b) (p<0.05).

The separate quantification of total free amino acids showed that Atlantic salmon lenses had an abundance of leucine, phenylalanine, glutamine and tyrosine that contributed to approx. 50% of the total free amino acid concentration, while NAH only contributed 5% of the total free amino acids (S4 Table). In rainbow trout lenses, NAH made up 32–40% of the total free amino acid concentration. The sum of free amino acids inclusive NAH in the lens was similar in both species and was not affected by temperature, with a mean value of 39 ±1 μmol g^-1^. Rainbow trout had significantly higher levels of N-acetylaspartate (NAA) compared to Atlantic salmon, however both species reared at 19°C had lower levels compared to 13°C (Fig 4). The level of NAA was reduced with increasing cataract score in Atlantic salmon.

**Fig 5:**
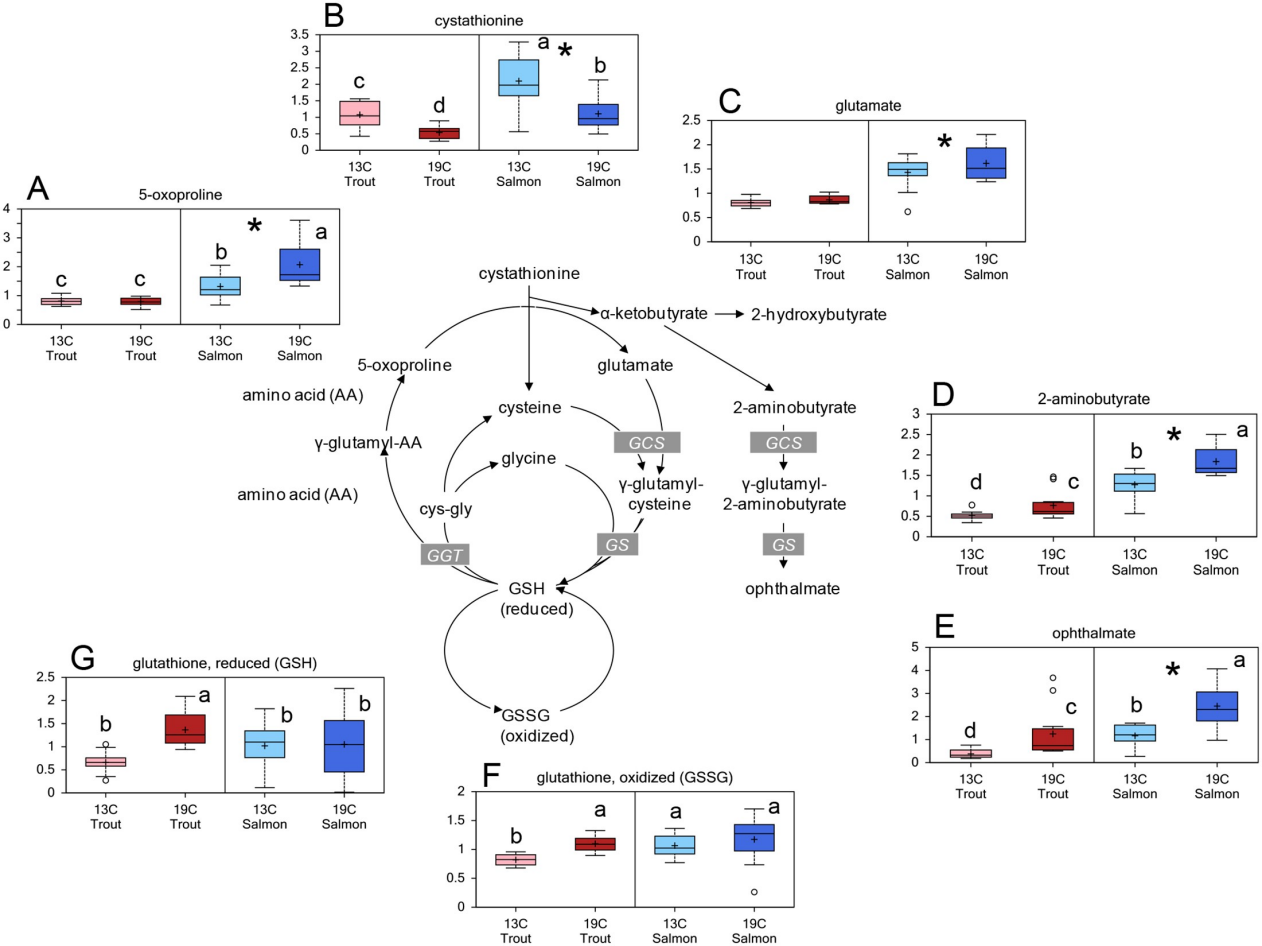
Lens GSH metabolism. Relative levels of metabolites related to GSH metabolism in lenses from Atlantic salmon and rainbow trout reared at 13 or 19°C at the end of the 35d experiment: (A) 5-oxoproline, (B) cystathionine, (C) glutamate, (D) 2-aminobutyrate, (E) ophtalmate, (F) oxidized glutathione (GSSG), (G) reduced glutathione (GSH). Significant differences between species are denoted by an asterisk (*), significant differences between temperatures and interaction effects are denoted by lower case letters (a, b) (p<0.05).

Several precursors and intermediates in the glutathione (GSH) and ophtalmate metabolism were present at significantly different levels in Atlantic salmon and rainbow trout lenses, and many were affected by temperature (Fig 5). The level of reduced glutathione (GSH) was higher in in rainbow trout reared at 19°C compared to 13°C, and a higher level in rainbow trout reared at 19°C compared to Atlantic salmon reared at 19°C. The level of oxidized GSH (GSSG) was significantly higher in trout reared at 19°C compared to 13°C. No differences were found in the level of cysteine, however the precursor cystathionine was present at significantly lower levels in both species reared at 19°C compared to 13°C, and higher in salmon compared to trout lenses. The GSH precursor glutamate and the break-down intermediate 5-oxoproline was higher in Atlantic salmon compared to rainbow trout, and higher salmon reared at 19°C than at 13°C. Salmon lenses had significantly higher levels of both ophtalmate and 2-aminobutyrate compared to rainbow trout lenses, and both species had higher levels in the fish reared at 19°C compared to 13°C.

**Fig 6:**
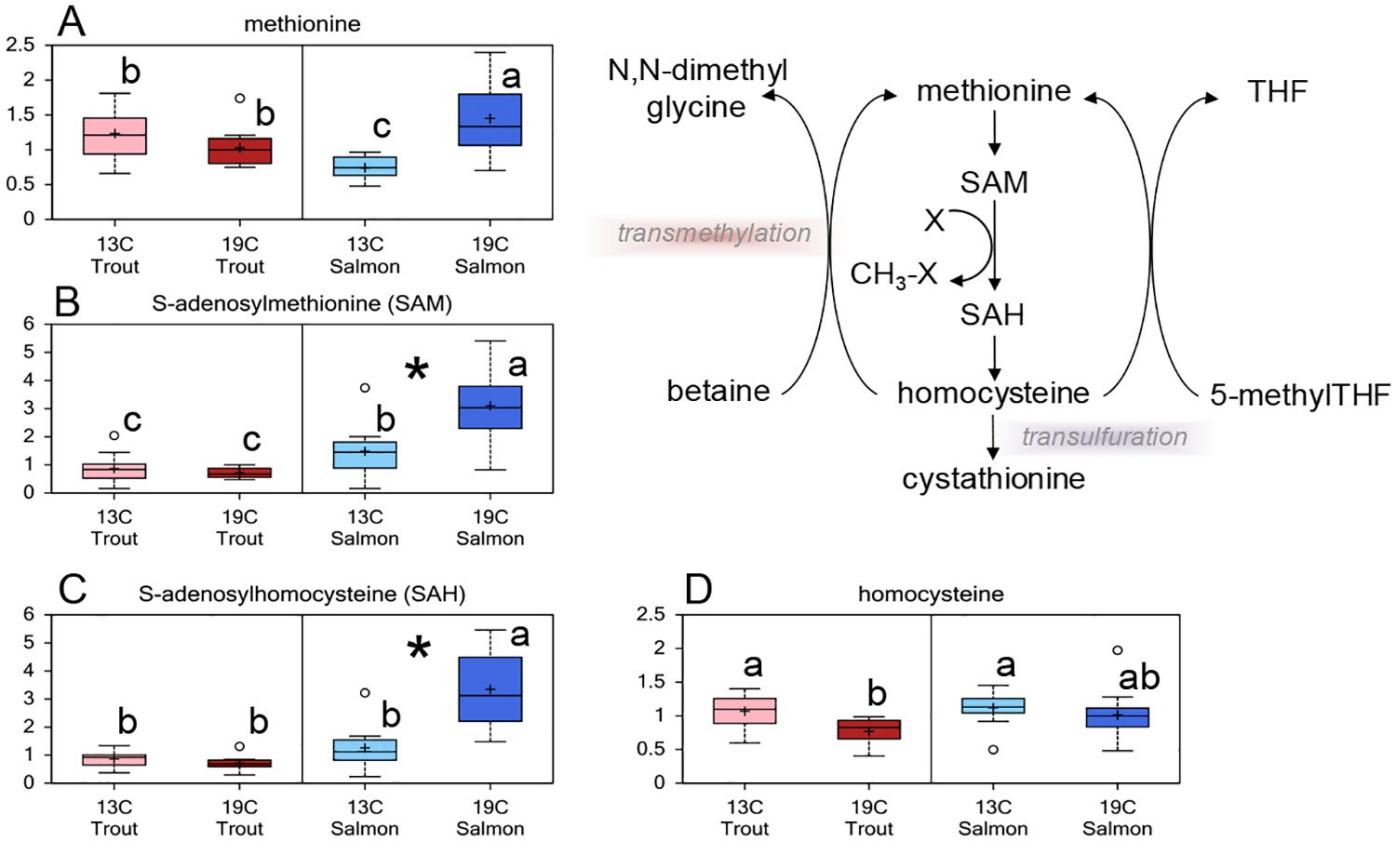
Lens 1-carbon metabolism. Relative levels of metabolites involved in the transmethylation pathway of the 1-carbon metabolism in lenses from Atlantic salmon and rainbow trout reared at 13 or 19°C at the end of the 35d experiment: (A) methionine, (B) S-adenosylmethionine (SAM), (C) S-adenosylhomocysteine (SAH), (D) homocysteine. Significant differences between species are denoted by an asterisk (*), significant differences between temperatures and interaction effects are denoted by lower case letters (a, b) (p<0.05).

Several intermediates in the transmethylation pathway of the 1-carbon metabolism were present at different levels in rainbow trout and Atlantic salmon, and Atlantic salmon reared at 19°C had significantly higher levels of S-adenosylmethionine (SAM) and S-adenosylhomocysteine (SAH) compared to salmon reared at 13°C (Fig 6).

A total of 26 peptides, 18 dipeptides and 8 gamma-glutamyl peptides, were present in the lenses (S1 Table). Of these, 15 dipeptides were present at higher levels in Atlantic salmon lenses compared to rainbow trout lenses, and the dipeptides that were affected by temperature were all found in lower levels in fish reared at 19°C compared to 13°C. The level of all gamma-glutamyl peptides differed between the species.

**Fig 7:**
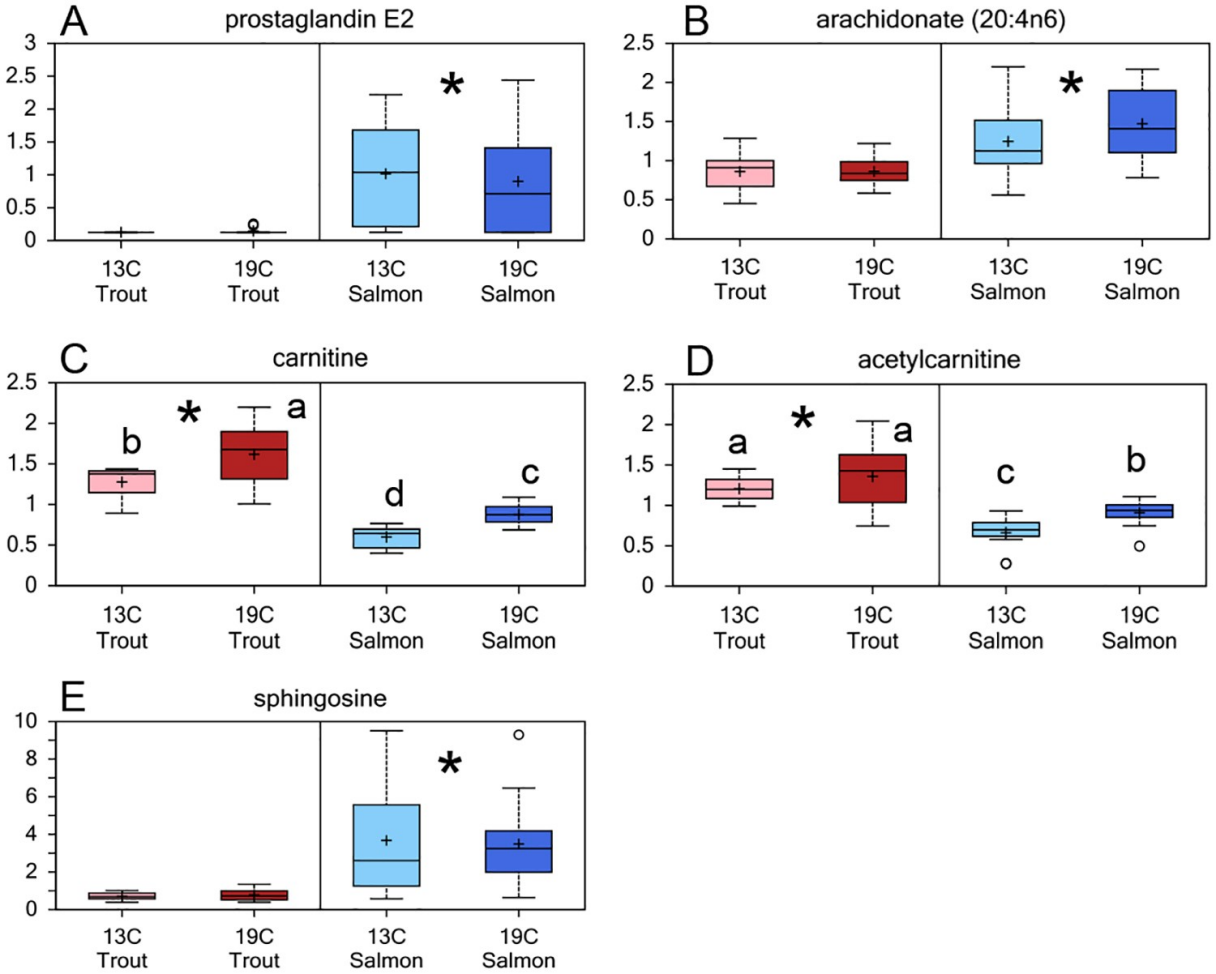
Lens lipid metabolism. Relative levels of metabolites related to the lipid metabolism in lenses from Atlantic salmon and rainbow trout reared at 13 or 19°C at the end of the 35d experiment: (A) prostaglandin E_2_, (B) arachidonate (20:4n-6), (C) carnitine, (D) acetylcarnitine, (E) sphingosine. Significant differences between species are denoted by an asterisk (*), significant differences between temperatures are denoted by lower case letters (a, b) (p<0.05).

Differences between the species were found for 24 of the 40 identified metabolites in the lipid pathway (S1 Table). Atlantic salmon lenses had significantly higher levels of arachidonic acid (ARA, 20:4n-6) compared to rainbow trout, and a significantly higher level of the ARA derived eicosanoid prostaglandin E_2_ (Fig 7). Atlantic salmon had a fivefold higher level of sphingosine compared to rainbow trout at both temperatures. Rainbow trout had higher levels of both carnitine and acetylcarnitine compared to Atlantic salmon, where carnitine was influenced by temperature for both species, while acetylcarnitine was only higher in salmon reared at 19°C compared to 13°C. Rearing temperature also affected the inositol metabolism, with higher levels of the inositol compounds, myo-, chiro- and scyllo-inositol in fish reared at 19°C compared to 13°C in both species, and higher levels in Atlantic salmon compared to rainbow trout at both temperatures (S1 Table).

**Fig 8:**
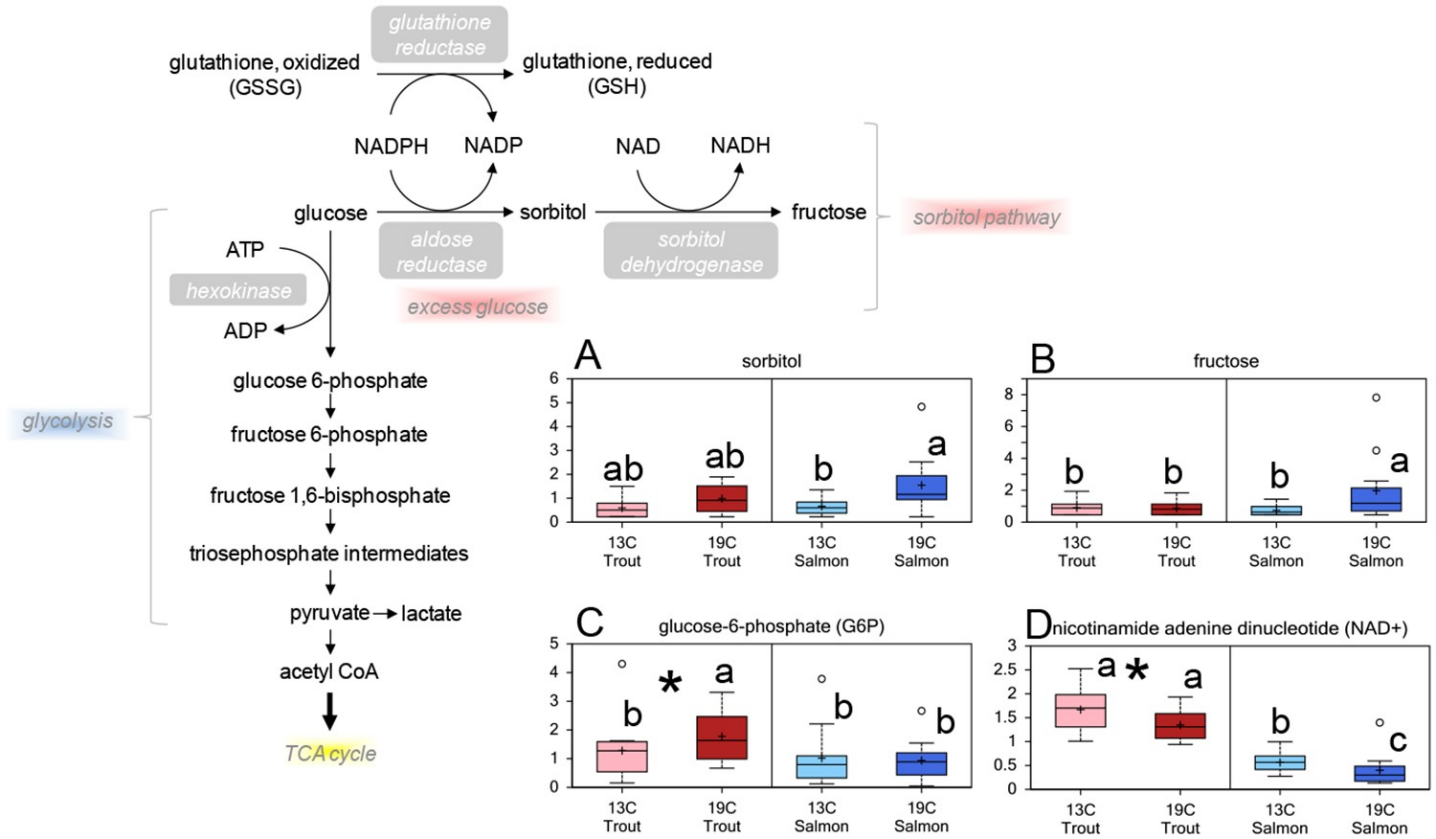
Lens carbohydrate metabolism. Relative levels of metabolites involved in glycolysis and the polyol pathway in lenses from Atlantic salmon and rainbow trout reared at 13 or 19°C at the end of the 35d experiment: (A) sorbitol, (B) fructose, (C) glucose-6-phosphate, (D) nicotinamide adenine dinucleotide (NAD+). Significant differences between species are denoted by an asterisk (*), significant differences between temperatures and interaction effects are denoted by lower case letters (a, b) (p<0.05).

The level of biochemicals in the carbohydrate metabolism differed between the species, with lower levels of glucose-6-phosphate (G6P) and higher levels of glucose-1-phosphate and lactate in Atlantic salmon compared to rainbow trout lenses (S1 Table). No differences were seen in the level of lens glucose between the species or at either temperature (S1 Table). Atlantic salmon lenses had a significantly higher level of sorbitol at 19°C compared to 13°C, and a similar trend was found in rainbow trout (p = 0.12) (Fig 8). Atlantic salmon reared at 19°C had a significantly higher lens fructose level compared to salmon reared at 13°C (Fig 8). A cataract associated effect was found in rainbow trout, with increased levels of fructose, glycerate and glucose in lenses from fish with cataracts compared to clear lenses (S3 Table).

All of the identified cofactors and vitamins differed between the species (S1 Table). Atlantic salmon had significantly lower levels of nicotinamide adenine dinucleotide (NAD+) (Fig 8), and both species had a lower level of NAD+ at 19°C compared to the respective fish reared at 13°C.

## Discussion

In the present study, cataract development in Atlantic salmon and rainbow trout reared at optimum and high temperature was investigated. The global metabolic profiles in lenses from Atlantic salmon and rainbow trout were characterized to investigate differences in lens metabolism between the species and study metabolic changes in the lenses caused by water temperature. In the present study, the cataract prevalence and the severity of cataracts were significantly higher in Atlantic salmon compared to rainbow trout despite equal farming and feeding conditions [3]. Both species had high growth rates at both temperatures, and this may have increased the susceptibility to cataract development [10, 11, 12]. The lens metabolic profile differed between the species, where several metabolites that are associated with cataract development were present at different levels in Atlantic salmon and rainbow trout. While these differences may be related to cataract severity, only a few of these were related to the cataract scores. Thus, the lens metabolic profiles likely indicate differences in lens metabolism that may explain or is the consequence of the higher susceptibility to cataracts for Atlantic salmon.

A major difference between the species was seen in the histidine metabolism, and independent quantification of histidine and NAH with reverse phase HPLC confirmed a higher concentration of both histidine and NAH in the rainbow trout compared to Atlantic salmon lenses. Sub-optimal levels of dietary histidine has been identified as a major risk factor for cataract development in farmed Atlantic salmon, and cataracts can be mitigated by increasing the dietary histidine concentration [7,19,20]. The cataract mitigating effect of dietary histidine has been attributed to the synthesis and concentration of NAH in the lens [20, 36]. The concentration of NAH in Atlantic salmon lenses has been shown to reflect the dietary histidine concentration, and a low level of NAH is therefore considered a marker for the risk for cataract development. In the present study, both species were fed the same diet, designed to cover the dietary amino acid requirements for growth for both species, with a dietary histidine concentration of 10 g His kg^-1^ feed. This concentration is however lower than the re-established requirement of 13.4 g His kg^-1^ feed to minimize the risk of cataract development for Atlantic salmon smolt after seawater transfer [20], and this may explain both the severity of cataracts and the almost depleted lens NAH concentration in Atlantic salmon in the present study. In comparison, the rainbow trout was able to maintain a higher lens NAH concentration, with lens NAH constituting approx. one third of the free amino acid pool, when given the same dietary histidine concentration. This clearly indicates differences in the metabolism as well as possibly also requirements for histidine between the species. The dietary histidine concentration was almost twice the estimated histidine requirement for growth for rainbow trout of 5–6 g His kg^-1^ dry matter [37], and this appeared to be sufficient to maintain a cataract preventive concentration of lens NAH. Thus, the observed difference in cataract development between the species may be explained by a different requirement or metabolism of histidine, with levels resulting in a higher susceptibility to cataracts for Atlantic salmon, regardless of rearing temperature.

NAH has been shown to function as an osmolyte in the fish lens thereby contributing to maintaining the water balance and volume regulation in the lens [21, 22]. The osmolality of the aqueous humour of the salmon eye appears to be coupled to the plasma level of ions, and have been found to be between 310 to 314 mOsm kg^-1^ in freshwater, and in the range 320 to 335 mOsm kg^-1^ in sea water [19]. Thus, the period after seawater transfer causes osmotic challenges for salmon smolts, and is considered to be a risk period for cataract development. The present study was performed with Atlantic salmon smolts and rainbow trout in seawater, and the depleted level of NAH in the salmon lens indicates that the lens’ ability to osmoregulate was lowered, while the high concentrations in the rainbow trout suggest a stronger standing defence against shifts in osmolality, and thus possibly a lower susceptibility to cataracts. The total concentration of free amino acids was similar in lenses from all groups, supporting the hypothesis that Atlantic salmon compensates with other amino acids to maintain homeostasis when there is not enough histidine available for NAH synthesis [19].

The Atlantic salmon lenses had significantly higher concentrations of the branched chain amino acids leucine, isoleucine and valine, and phenylalanine compared to rainbow trout. Branched amino acids are traditionally connected to oxidative metabolism and energy production in muscle tissue [38]. Degradation of branched amino acids may provide acyl-CoA derivatives [39] for the purpose of energy supply or substrate for acetylation reactions, especially in the lens epithelial cell layers that are metabolically active with mitochondria. In human epidemiological research using plasma metabolic profiling, branched and aromatic amino acids were identified as highly significant predictors of future diabetes [40]. The consequences of a high concentration of these branched amino acids in the salmon lens is unknown, however intermediates in the metabolism of these amino acids may induce oxidative damage [41], thus in itself increase the risk of cataract development. The global metabolic profile also indicated differences in the levels of other acetylated amino acids than NAH between the species, which may be explained by a species specific difference in the acetylation of amino acids, or reflect a compensation mechanisms to trap other amino acids at low histidine and NAH levels in Atlantic salmon lenses compared to rainbow trout lenses. The levels of dipeptides and gamma-glutamyl amino acids support the indication of differences in lens protein and amino acid metabolism between species and as a response to high temperature, and may indicate a higher level of degraded proteins in the lenses and transportation of amino acids across the cell membrane in fish reared at 19°C compared to 13°C.

The concentration of total lipids in Atlantic salmon lenses is approximately 5 mg g^-1^ wet weight and the lipid class composition indicates that the main function of lens lipids are for structural purposes and messengers, as opposed to energy, with an abundance of phospholipids and cholesterol [23]. In the present study, Atlantic salmon lenses had a higher level of sphingosine, an intermediate in the ceramide synthesis, compared to rainbow trout, and this may be related to the higher severity of cataracts in the salmon. Ceramide has many physiological functions beyond being a component in the membrane structure, among others in signalling, cell functions and apoptosis [42]. High levels of ceramide have been found in human cataractous lenses [43], and have been suggested to be part of the aetiology of human age-related cataracts [44].

The Atlantic salmon lenses had a higher level of non-esterified arachidonic acid (20:4n-6, ARA) and prostaglandin E_2_ (PGE_2_) compared to rainbow trout. This may be a result of the more severe damage to the Atlantic salmon lenses, resulting in a release of ARA from the cell membranes and thus a higher production of PGE_2_, as shown in cultured rabbit epithelial cells damaged by UVB-radiation [45]. Prostaglandins are involved in several biological functions that may influence cataract formation such as regulation of cell proliferation [45], inflammation processes in lens epithelial cells from cataract patients [46] and osmoregulation [47]. Thus, the higher levels in Atlantic salmon compared to rainbow trout may be related to the higher severity of cataracts in salmon, however the exact mechanism or cause-effect relationship is not known.

Cataract development is hypothesized to be related to elevated water temperatures, however it is not clear whether this relates to cell growth or metabolic costs [7]. Since feed intake and growth rate was not affected by the rearing temperature in the present study, the observed differences in the metabolic profile in lenses from fish reared at 13 and 19°C are interpreted as metabolic differences caused by the different rearing temperatures.

Osmotic cataracts in Atlantic salmon are usually associated with reduced osmoregulatory abilities after seawater transfer and is characterised by a more permeable membrane, leading to a rapid water uptake, swelling and ruptures in the lens basal membrane [1]. The level of N-acetylaspartate (NAA), a suggested analogue osmolyte to NAH in the brain [48, 49] was present at lower levels in both rainbow trout and Atlantic salmon reared at 19°C compared to 13°C. Together with the reduced concentration of NAH in the rainbow trout lenses reared at high temperature this suggests an increased usage of osmolytes at high temperature. In addition, the Atlantic salmon lenses had lower levels of NAA in lenses with a high cataract score, supporting a connection between the lens’ ability to osmoregulate and cataract formation.

Osmotic cataracts have also been associated with elevated plasma glucose concentrations in Atlantic salmon [26]. In the present study, the plasma glucose concentration was significantly higher in Atlantic salmon reared at 19°C compared to 13°C [3]. Although no differences were observed in the lens glucose levels at the end of the experiment, differences observed in intermediates in the glucose breakdown pathways suggest a temperature dependent dysfunction or an overload of glycolysis in the Atlantic salmon lenses. The higher level of sorbitol in Atlantic salmon lenses reared at 19°C may have resulted in osmotic stress and consequently contributed to cataract formation, as seen in hyperglycaemic animals and humans [50, 51]. The level of fructose and glucose increased with increasing cataract score in rainbow trout lenses, suggesting that alterations in the glucose metabolism may be related to the observed cataract development in rainbow trout. Both species had increased levels of metabolites in the myo-inositol biosynthetic pathway in the high temperature groups, indicating that temperatures affect the carbohydrate metabolism in both species. Altogether, the results from the present study suggest that temperature mediated alterations in the carbohydrate metabolism may be associated with ongoing cataractogenesis in both species.

Due to the low degree of protein turnover, the lens is very susceptible to post-translational modifications (like glycation) that results in an alteration of structural proteins. In the present study, elevated water temperature resulted in a higher fructose level in the Atlantic salmon lenses, which is consistent with a lower level of NAD, a cofactor for sorbitol dehydrogenase. Fructose is a potent glycation agent, thus contributing to the formation of advanced glycation end-products (AGEs) [52] that cause conformational changes and increase cross-linking in the lens [53]. Glycation has been shown to be involved in the cataractogenesis of hyperglycemic and diabetic animals [54]. Rainbow trout may have stronger defence against cross-linking compared to Atlantic salmon due to higher levels of acetylcarnitine that has been shown to prevent glycation of crystallins, possibly through post-translational acetylation of the glycation sites [55] or through effects on apoptosis regulating genes [56]. The higher lens level of histidine in rainbow trout may also be beneficial as histidine has been shown to exhibit anti-cross linking activity [57], and inhibit the complications of glucose-induced oxidation and glycation, as shown in diabetic mice [58]. Carnosine has been shown to delay the progression of cataracts caused by glycation in diabetic rats [59], however, the proposed imidazole analogue in salmonid lenses NAH does not have an anti-cross linking activity [57]. The higher levels of metabolites in the 1-carbon metabolism in Atlantic salmon reared at 19°C may also have consequences for the post-translational modification of proteins, whereas SAM can methylate lens proteins [60]. The methylation of cysteine residues in crystallins has been suggested to protect the lens from disulfide bonding and cross-linking [61], however, the changes may be age related, as no differences in methylation level have been found between healthy and cataractous lenses [60].

GSH is an important innate antioxidant in the lens, and a low concentration is associated with cataract development in animals and humans [62, 63, 64, 65]. As a response to oxidative stress, cellular GSH levels are expected to be lowered, as found in cataractous lenses, both *in vivo* [28,66] and experimentally induced cataract [23,63,65]. During changes in temperature, the cellular metabolism is reorganised in fish, and the activity of GSH dependent enzymes have been shown to increase in gold fish tissues, along with higher levels of thiols such as GSH [16]. This may explain the higher level of both reduced glutathione (GSH) and oxidized glutathione disulfide (GSSG) in rainbow trout reared at 19°C compared to rainbow trout reared at 13°C, indicating that the rainbow trout were able to increase the defence against oxidative stress in the lens and thereby possibly maintain the redox homeostasis in the lenses. In comparison, the Atlantic salmon lenses had large variations in the lens GSH level at both temperatures; indicating large individual differences in the GSH metabolism and the ability to regenerate GSH, resulting in a lower ability to withstand oxidative stress.

Ophtalmate, a structural analogue of GSH [67], was present at higher levels in both Atlantic salmon and rainbow trout exposed to 19°C, and at higher levels in Atlantic salmon compared to rainbow trout. Ophtalmate has been suggested to be a biomarker for hepatic GSH depletion in humans, and thus a marker for oxidative stress [68]. Assuming that ophtalmate represents a marker for GSH depletion in salmonid lenses, the present results indicate a higher oxidative pressure in Atlantic salmon lenses compared to rainbow trout lenses, and that elevated temperatures resulted in oxidative stress in lenses from both species. From the present results, it is suggested that ophtalmate may be a useful marker to assess oxidative stress in lenses.

The differences in lens oxidative status between the species may also be due to the higher lens concentration of NAH in rainbow trout compared to the Atlantic salmon. The histidine containing imidazole carnosine appears to have an antioxidant function in the canine and human lenses [66] and thus mitigate cataract development. These dipeptide imidazoles are not found in the salmon lens [19], while lens NAH has been proposed to cover the analogue antioxidant function in the salmon lens [23]. The latter *ex vivo* study on Atlantic salmon lenses showed that enzymes in the GSH system are affected by additional histidine in the medium, by up-regulating of genes encoding glutaredoxin and GPx4 [23], and histidine may therefore directly affect the innate antioxidant system in salmon lenses. Thus, the reduction in lens NAH concentration at high temperature seen in rainbow trout lenses may be because NAH was used as an antioxidant, in addition to the osmolyte function. For the same reason, the higher level of free histidine found in rainbow trout lenses compared to Atlantic salmon lenses may also have contributed to the antioxidant defence [69].

In summary, Atlantic salmon was more susceptible to cataract development than rainbow trout under the given similar rearing conditions. The difference in histidine metabolism may explain the apparent difference in cataract susceptibility between the species. However, almost 50% of the rainbow trout developed cataract at both temperatures, suggesting that other factors may be involved in the aetiology of cataracts in rainbow trout, such as the observed effects in the carbohydrate metabolism. Rearing temperature did not increase the cataract development significantly in either species during the 35-day experiment; however, the metabolic profile indicates that high temperature alters the osmoregulatory ability, carbohydrate metabolism and redox regulation in the lenses, which may result in a higher risk for cataract development.

## Supporting information

S1 Table Lens biochemical heat map. Heat maps showing the identified biochemicals in Atlantic salmon and rainbow trout lenses reared at 13 or 19°C. Red colour indicates a significantly higher level and green colour indicates a significantly lower level of the metabolite (contrasts) (*p*<0.05).
(XLSX)

S2 Table Atlantic salmon lens metabolites in relation to cataract scores. Biochemicals that were significantly different in lenses with different cataract scores in Atlantic salmon lenses. The data are presented as mean values of each metabolite per cataract score 1–3. Red colour indicates a significantly higher level and green colour indicates a significantly lower level of the metabolite (contrasts) (*p*<0.05).
(XLSX)

S3 Table Rainbow trout lens metabolites in relation to cataract scores. Biochemicals that were significantly different in lenses with different cataract scores in rainbow trout lenses. The data are presented as mean values of each metabolite per cataract score 0–2. Red colour indicates a significantly higher level and green colour indicates a significantly lower level of the metabolite (contrasts) (*p*<0.05).
(XLSX)

S4 Table Free amino acid concentration in Atlantic salmon and rainbow trout lenses. The concentration of amino acids in lenses from Atlantic salmon and rainbow trout reared at 13 or 19°C at the end of the 35 days experiment. Significant differences are indicated by the p-values in each column.
(XLSX)
